# Work addiction, work engagement, job burnout, and perceived stress: A network analysis

**DOI:** 10.3389/fpsyg.2023.1130069

**Published:** 2023-03-29

**Authors:** Piotr Bereznowski, Paweł Andrzej Atroszko, Roman Konarski

**Affiliations:** Faculty of Social Sciences, Institute of Psychology, University of Gdańsk, Gdańsk, Poland

**Keywords:** network analysis, job burnout, perceived stress, work addiction, work engagement, workaholism, network approach

## Abstract

**Introduction:**

Recently, the network theory of mental disorders has been used to conceptualize work addiction as a dynamic system of symptoms in direct relationships. This study aimed to extend previous work by investigating the direct relationships of work addiction symptoms with dimensions of work engagement, job burnout, and perceived stress.

**Methods:**

These phenomena were measured with the Bergen Work Addiction Scale, the Utrecht Work Engagement Scale, the Maslach Burnout Inventory–General Survey, and the Perceived Stress Scale. The sample comprised 676 working Poles with a mean age of 36.12 years (SD = 11.23). The network analysis followed the guidelines for estimating psychological networks from cross-sectional data.

**Results:**

Work engagement and job burnout were more closely associated with each other than with work addiction which supports the notion that engagement and burnout represent polar opposites of the same construct and that work addiction is a separate phenomenon, related to both work engagement and job burnout *via* specific pathways. The symptoms of work addiction were connected with other phenomena through four direct relationships: (1) mood modification—absorption, (2) mood modification—stress, (3) withdrawal—absorption, and (4) problems—exhaustion.

**Discussion:**

These findings narrow down and specify hypotheses regarding potential mechanisms leading from engagement to addiction and from addiction to burnout. The possible mechanisms focus on the absorption component and mood modification related to efforts focused on alleviating chronic stress and negative emotional states. In turn, problems arising from work addiction may lead to exhaustion. Future studies investigating these mechanisms in detail may enable proper prevention programs and therapeutic interventions.

## 1. Introduction

Over 745 thousand deaths worldwide could be attributed to overworking every year, and it only accounts for cardiovascular-related problems attributed to workload (Pega et al., 2021). A substantial portion of these deaths could be arguably attributed to compulsive overworking, which is strictly related to excessively high workload. Compulsive overworking has a prevalence rate from 8.3 to 20.6% in nationally representative samples of working populations, depending on the country (for an overview of prevalence data, see Atroszko, 2022a) and is gradually recognized as a major epidemiological concern globally (compulsive overworking is often referred to as work addiction or workaholism; Griffiths et al., 2018; Balducci et al., 2020; Atroszko, 2022a,b). Meta-analyses and reviews suggest that compulsive overworking could lead to occupational stress, deteriorated work engagement, job burnout, and eventually, a global burden of disease (Patel, 2011; Clark et al., 2016; Di Stefano and Gaudiino, 2019; Atroszko et al., 2020a). In this study, we aimed to investigate the direct relationships of work addiction symptoms to dimensions of work engagement, job burnout, and perceived stress.

Several conceptualizations of compulsive overworking are present in the literature (e.g., Schaufeli et al., 2009; Vallerand et al., 2010; Snir and Harpaz, 2012; Loscalzo and Giannini, 2018; Atroszko et al., 2019; Clark et al., 2020), including the one using a behavioral addictions framework and labeling this phenomenon “work addiction” (for an overview, see Atroszko, 2022a,b). Work addiction has been defined as “a compulsion to work and preoccupation with work activities leading to a significant harm and distress of a functionally impairing nature to the individual and/or other significantly relevant relationships (friends and family). The behavior is characterized by the loss of control over the working activity and persists over a significant period of time. This problematic work-related behavior can have varying intensity from mild to severe” (Atroszko et al., 2019, p. 9). The work addiction conceptualization often refers to the common addiction components model, which includes seven addiction symptoms (Brown, 1993; Griffiths, 2005): (1) *salience* (work dominates thoughts, feelings, and behavior), (2) *tolerance* (increasing amounts of work are needed to achieve former mood modification effects), (3) *mood modification* (work leads to feelings of “high” or “escape”), (4) *relapse* (repeated revisions to a pattern of excessive work), (5) *withdrawal* (being unable to work leads to unpleasant feelings and/or states), (6) *conflict* (with other activities, needs, and people), (7) *problems* (health and/or other problems resulting for excessive work; Griffiths, 2011; Andreassen et al., 2012).

Recently, the network theory of mental disorders (Borsboom, 2017) has been used to conceptualize work addiction as a network of symptoms in direct relationships (Bereznowski et al., 2021, 2022). A network is a graph composed of nodes representing observed variables (e.g., a symptom of work addiction or a dimension of job burnout) and edges representing the estimated strength of direct relationships between the observed variables. Positive edges are usually depicted as solid blue lines and indicate that higher (lower) levels of node A co-occur with higher (lower) levels of node B when levels of other nodes in a network remain constant. Negative edges are usually depicted as solid red lines and indicate that higher (lower) levels of node A co-occur with lower (higher) levels of node B when levels of other nodes in a network remain constant. The magnitude of similarity of the levels of the two nodes depends on the strength of the relationships between a pair of nodes. A process of analyzing a pattern of the estimated edge weights, using specially designed statistical methods, is called network analysis (Epskamp et al., 2018). The results of network analysis could provide valuable insights regarding potential targets of therapeutic interventions and prevention programs (the target could be either a node or an edge between nodes; Cramer et al., 2016; Borsboom, 2017).

So far, there have been two studies using the network theory framework to investigate direct relationships of symptoms of work addiction (Bereznowski et al., 2021; Bereznowski et al., 2022; both studies used the same samples (four and three samples, respectively) and one of these samples was used in the present study as well). Bereznowski et al. (2022) investigated the simplest possible network which included only the symptoms of work addiction. The results showed that the symptoms of work addiction formed two clusters. The first cluster included silence, mood modification, and withdrawal. The second cluster included tolerance, relapse, conflict, and problems. These clusters showed partial overlap with a distinction between peripheral (cognitive salience, tolerance, and euphoria) and core (conflict, withdrawal symptoms, relapse and reinstatement, and behavioral salience) symptoms of gaming addiction (Charlton and Danforth, 2007). Based on these results, Bereznowski et al. (2021) argued that the inclusion of other work-related phenomena might help validate the claim that the two clusters represent more and less pathological groups of work addiction symptoms.

Bereznowski et al. (2021) investigated the relationships between symptoms of work addiction and dimensions of work engagement. Work engagement is a positive work-related mental state. According to the most popular conceptualization (Schaufeli et al., 2002), it is characterized by (1) *vigor* (”high levels of energy and mental resilience while working, the willingness to invest effort in one’s work, and persistence even in the face of difficulties”; Schaufeli et al., 2002, p. 74), (2) *dedication* (”experiencing a sense of significance, enthusiasm, inspiration, pride, and challenge”; Schaufeli et al., 2002, p. 74), and (3) *absorption* (”being fully concentrated and deeply engrossed in one’s work, whereby time passes quickly and one has difficulties with detaching oneself from work”; Schaufeli et al., 2002, p. 75). As work engagement is a positive phenomenon, it was expected that the three dimensions of work engagement should have stronger positive relationships with less pathological than with more pathological symptoms of work addiction. The results showed that dimensions of work engagement formed an independent cluster which was mainly connected with symptoms of work addiction through positive relationships with the absorption component (the strongest relationships were observed with mood modification and withdrawal). However, there were also negative connections between vigor and mood modification, conflict, and problems, and between dedication and mood modification which could indicate negative consequences of work addiction on work engagement (Bereznowski et al., 2021), which might be mediated by job burnout (work addiction leads to job burnout which in turn deteriorates work engagement).

The purpose of this study was to extend the previous work of Bereznowski et al. (2021, 2022) by including in the network two negative phenomena: job burnout (a possible mediator between work addiction and work engagement) and perceived stress (one of the most important causes of work addiction). In the 11th Revision of the International Classification of Diseases (ICD-11; World Health Organization, 2019), job burnout has been classified as an occupational phenomenon (a reason for which people contact health services other than illness). Job burnout has been defined as a result of chronic workplace stress that has not been successfully managed, which is characterized by (1) *exhaustion* (feelings of energy depletion), (2) *cynicism* (increased mental distance from one’s work and negativism related to one’s work), and (3) *professional efficacy* (a sense of ineffectiveness and lack of accomplishment; World Health Organization, 2019). Perceived stress has been defined as “the feelings or thoughts that an individual has about how much stress they are under at a given point in time or over a given time period” (Phillips, 2013, p. 1453). When investigating the extended network of work addiction, we focused on the following research questions. Do job burnout and perceived stress form independent clusters in the network? Does the inclusion of job burnout and perceived stress in the network influence direct relationships between symptoms of work addiction and dimensions of work engagement? Which symptoms of work addiction are directly related to perceived stress? Which symptoms of work addiction are directly related to which dimensions of job burnout?

Previous research grounded in the network framework showed that work addiction symptoms form two clusters possibly representing core and peripheral symptoms of addiction and that dimensions of work engagement form the third independent cluster (Charlton and Danforth, 2007; Bereznowski et al., 2021, 2022). Based on the previous research, it seems reasonable to assume that the dimensions of job burnout form another independent cluster in the network. Consequently, we expect that work addiction symptoms, work engagement dimensions, and job burnout dimensions will form four clusters in the estimated network: (1) more pathological (i.e., core) work addiction symptoms (tolerance, relapse, conflict, and problems), (2) less pathological (i.e., peripheral) work addiction symptoms (salience, mood modification, and withdrawal), (3) work engagement dimensions, and (4) job burnout dimensions. After considering the crucial role of perceived stress in both work addiction and job burnout, particularly its exhaustion component (Bianchi and Schonfeld, 2018; Griffiths et al., 2018; World Health Organization, 2019), we expect stress to be directly related to relevant components of work addiction and job burnout.

A study by Bereznowski et al. (2021) grounded in the network framework, showed that each work engagement dimension had direct relationships with at least two symptoms of work addiction. However, most of these relationships were very weak. Meta-analyses grounded in the latent trait framework showed that work addiction was associated with a single dimension of work engagement (i.e., absorption; Clark et al., 2016; Di Stefano and Gaudiino, 2019). Based on these premises, we predict that after controlling for the effects of job burnout dimensions and perceived stress, it is likely that the only statistically significant relationships between work addiction symptoms and work engagement dimensions will be the relationships including absorption.

Previous meta-analyses showed that work addiction was associated with stress and other stress-related psychological phenomena such as job demands, work-life balance, or work-family conflict (Patel, 2011; Clark et al., 2016; Atroszko, 2022a,b). Based on the definitions of the symptoms of work addiction, only four out of seven work addiction symptoms should be related to perceived stress. Two symptoms might be related directly (i.e., mood modification and withdrawal), and two indirectly (conflict and problems). Based on these premises, we predict that only mood modification and withdrawal will have direct relationships with perceived stress.

Finally, a meta-analysis conducted by Clark et al. (2016) showed that work addiction was associated with all dimensions of job burnout. However, some work addiction symptoms might be more pathological (tolerance, relapse, conflict, and problems) than others (salience, mood modification, and withdrawal; Bereznowski and Konarski, 2020; Bereznowski et al., 2021; see also Charlton and Danforth, 2007). Consequently, we predict that only these more pathological symptoms (i.e., tolerance, relapse, conflict, and problems) will have direct relationships with the dimensions of job burnout.

**FIGURE 1:**
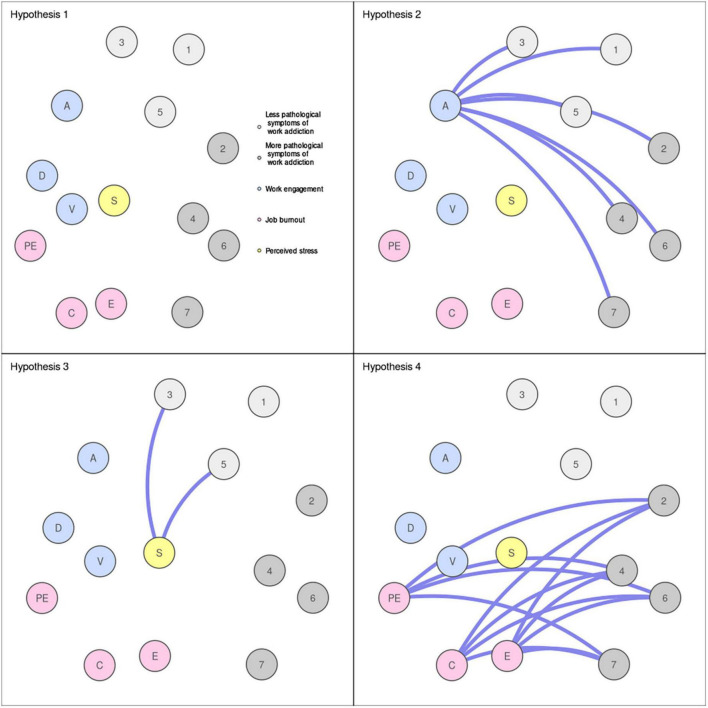
The visual representation of this study’s hypotheses in a generic form. The panel for hypothesis 1 presents the hypothesized clusters of symptoms. The panels for hypotheses 2, 3, and 4 present the hypothesized direct relationships between work addiction symptoms and dimensions of other phenomena in the network. 1 = salience; 2 = tolerance; 3 = mood modification; 4 = relapse; 5 = withdrawal; 6 = conflict; 7 = problems. S, perceived stress; E, exhaustion; C, cynicism; PE, professional efficacy; V, vigor; D, dedication; A, absorption.

Based on previous empirical research and theoretical considerations, we formulated the following hypotheses. Hypothesis 1: The network including work addiction, work engagement, job burnout, and perceived stress will have four clusters of nodes (cluster 1: tolerance, relapse, conflict, and problems; cluster 2: salience, mood modification, and withdrawal; cluster 3: work engagement dimensions; cluster 4: job burnout dimensions). Hypothesis 2: Work addiction symptoms will have direct relationships only with one dimension of work engagement (i.e., absorption). Hypothesis 3: Only mood modification and withdrawal will have direct relationships with perceived stress. Hypothesis 4: Only tolerance, relapse, conflict, and problems will have direct relationships with job burnout dimensions. For a visual representation of the hypotheses see Figure 1. Please note that Figure 1 presents the hypotheses in a generic form and that it is not required to observe all the edges presented in Figure 1 to confirm hypotheses 2 and 4.

## 2. Materials and methods

### 2.1. Study design

Data collection was based on convenience sampling of working professionals in Poland and took place from January 2014 to July 2016 (this sample was gathered during the research with a focus on work addiction; Atroszko et al., 2017). The study was a pen-and-pencil cross-sectional study. Working individuals from a wide range of professions (e.g., managers, lawyers, academics, medical doctors, teachers, engineers, accountants, IT specialists, and functionaries) were invited directly or through their employers to participate in the study. The participants filled in the questionnaire in an individual setting during their working hours (a few employers decided to gather the willing employees in a conference room for the time of filling in the questionnaire). Participation in the study was completely anonymous, and no monetary or other material rewards were offered.

### 2.2. Ethics

The study was reviewed and approved by the Norwegian Data Protection Official for Research and the Research Ethics Committee at the Institute of Psychology of the University of Gdańsk in Poland. Written informed consent was obtained from each participant before the completion of the questionnaire.

### 2.3. Participants

Initially, the sample included responses from 723 working Poles. After listwise deletion, the sample included 676 individuals. Their mean age was 36.12 years (SD = 11.23), ranging from 20 to 79. Detailed sociodemographic characteristics of the sample after listwise deletion of observations with missing data on work addiction, work engagement, job burnout, and perceived stress are presented in Table 1.

**TABLE 1:** Sociodemographic characteristics of the sample.

Variable	*n* (%)/*M* (SD)/range
**Sex**	
Female	476 (70.4%)
Male	191 (28.3%)
No answer	9 (1.3%)
Age (*M* [SD])	36.12 (11.23)
Age (range)	20–79
**Marital status**	
In a relationship	529 (78.3%)
Not in a relationship	143 (21.2%)
No answer	4 (0.6%)
**Number of children**	
0	282 (41.7%)
1	147 (21.7%)
2	147 (21.7%)
3	31 (4.6%)
4 or more	14 (2.1%)
No answer	55 (8.1%)
Years of education (*M* [*SD*])	17.90 (2.86)
Years of education (range)	9–26
Years of education (no answer)	9 (1.3%)
**Managerial position**	
Non-managerial	424 (62.7%)
Lower managerial	107 (15.8%)
Middle managerial	54 (8.0%)
Higher managerial	39 (5.8%)
No answer	52 (7.7%)
Working hours per week (*M* [SD])	45.64 (11.81)
Working hours per week (range)	4–98
**Work status**	
Full-time worker	602 (89.1%)
Part-time worker	58 (8.6%)
No answer	16 (2.4%)
Gross income (*M* [SD])^a^	45744.72 (32692.02) PLN
Gross income (range)^a^	0–200000 PLN

^a^Past year’s personal annual income before tax in Polish currency (i.e., PLN).

### 2.4. Measures

Participants were asked about their basic demographic information (sex, age, marital status, and years of education) and basic information about their work [level of managerial position, working hours per week, work status, and an open-ended question about their last year’s income (i.e., past year’s personal annual income before tax)].

#### 2.4.1. Work addiction

The Bergen Work Addiction Scale (BWAS; Andreassen et al., 2012) consists of seven items based on the seven symptoms of addiction (e.g., “How often during the last year have you thought of how you could free up more time to work?”; Brown, 1993; Leshner, 1997; Griffiths, 2005). Each item asks respondents how often they experienced a given symptom during the past 12 months. The responses are provided on a 5-point Likert scale ranging from 1 (*never*) through 2 (*rarely*), 3 (*sometimes*), 4 (*often*) to 5 (*always*). This measure does not have a skip-structure, and we did not preprocess the obtained responses in any way. The Polish version of the BWAS showed good content, convergent, criterion, and factor validity in previous studies (Atroszko et al., 2017). In this study, the Cronbach’s alpha reliability coefficient was 0.84.

#### 2.4.2. Work engagement

The 9-item version of the Utrecht Work Engagement Scale (UWES-9; Schaufeli et al., 2006) consists of three items for each dimension of work engagement: vigor (e.g., “At my work, I feel bursting with energy.”), dedication (e.g., “I am enthusiastic about my job.”), and absorption (e.g., “I am immersed in my work.”). Each item asks respondents how often they experienced a described state during their lifetime. The responses are provided on a 7-point Likert scale ranging from 1 (*never*) through 2 (*a few times a year or less*), 3 (*once a month or less*), 4 (*a few times a month*), 5 (*once a week*), 6 (*a few times a week*) to 7 (*everyday*) and summed up for each dimension. This measure does not have a skip-structure, and we did not preprocess the obtained responses in any other way than obtaining a sum for each dimension. The UWES showed good content, convergent, criterion, and factor validity in previous studies (Schaufeli et al., 2006; for a systematic review of research in Polish context, see Pollak et al., 2017). In this study, the Cronbach’s alpha reliability coefficients were 0.85 (vigor), 0.83 (dedication), and 0.79 (absorption).

#### 2.4.3. Job burnout

The Maslach Burnout Inventory – General Survey (MBI-GS; Maslach and Jackson, 1981) consists of 16 items, five for exhaustion (e.g., “I feel burned out from my work”), five for cynicism (e.g., “I have become less enthusiastic about my work”), and six for professional efficacy (e.g., “I feel confident that I am effective at getting things done “; higher scores indicate lower levels of job burnout). Each item asks respondents how often they experienced a described state during their lifetime. The responses are provided on a 7-point Likert scale ranging from 1 (*never*) through 2 (*a few times a year or less*), 3 (*once a month or less*), 4 (*a few times a month*), 5 (*once a week*), 6 (*a few times a week*) to 7 (*everyday*) and summed up for each dimension. This measure does not have a skip-structure, and we did not preprocess the obtained responses in any other way than obtaining a sum for each dimension. The MBI-GS showed good content, convergent, criterion, and factor validity in previous studies (e.g., Schutte et al., 2000; for results of research in Polish context, see Chirkowska-Smolak and Kleka, 2011; Golonka et al., 2019). In this study, the Cronbach’s alpha reliability coefficients were 0.89 (exhaustion), 0.71 (cynicism), and 0.83 (professional efficacy).

#### 2.4.4. Perceived stress

The 10-item version of the Perceived Stress Scale (PSS-10; Cohen et al., 1983) measures a general dimension of perceived stress. Each item asks respondents how often they experienced a described state during the past month (e.g., “In the last month, how often have you felt nervous and stressed?”). The responses are provided on a 5-point Likert scale ranging from 1 (*never*) through 2 (*almost never*), 3 (*sometimes*), and 4 (*fairly often*) to 5 (*very often*) and summed up for the general dimension of perceived stress. This measure does not have a skip-structure, and we did not preprocess the obtained responses in any other way than obtaining a sum for the general score. The Polish version of the PSS-10 showed good content, convergent, criterion, and factor validity in previous studies (Lee, 2012, for results of research in Polish context, see Juczyński and Ogińska-Bulik, 2009), including good criterion validity of the current version (Atroszko et al., 2020b). In this study, the Cronbach’s alpha reliability coefficient was 0.84.

### 2.5. Statistical analyses

All analyses were carried out with R version 4.0.5 (R Core Team, 2021) and visualized with the qgraph 1.6.9 package (Epskamp et al., 2012). For reporting the results, we followed the reporting standards for psychological network analyses in the cross-sectional data set by Burger et al. (2022). The analytic code for all analyses performed in this study and the Supplementary material are available at https://osf.io/jvqfa/.

#### 2.5.1. Network estimation

We estimated the network using the bootnet 1.4.7 package (Epskamp et al., 2018) and the EBICglasso method with the threshold parameter equal to TRUE. A layout for visualizations was obtained by setting the layout parameter to “spring.” To search for clusters of nodes, we used a spin-glass algorithm implemented in the igraph 1.2.6 package (Csardi and Nepusz, 2006).

#### 2.5.2. Network stability

To investigate the stability of the network, we used the bootnet 1.4.7 package (Epskamp et al., 2018), using non-parametric bootstrapping and case bootstrapping based on 1,000 bootstrap samples. As a measure of network stability, we used the correlation stability coefficient, which represents the maximum proportion of cases that can be dropped, such that with 95% probability, the correlation between original centrality measures and centrality of networks based on subsets is 0.70 or higher. A correlation stability coefficient higher than 0.50 is regarded as an indicator of good stability, and a correlation stability coefficient higher than 0.25 is regarded as an indicator of acceptable stability (Epskamp et al., 2018).

#### 2.5.3. Network inference

We estimated node centrality based on node strength. A standard version of the node strength is a metric equal to the sum of absolute values of all edges of a given node to all other nodes. Its lowest possible value is 0.00 (a node is not connected). Its highest possible value is the number of nodes in a network minus one (a node is connected with all nodes in a network with edges of magnitude 1.00). The nodes with the above-average standard version of the node strength can be identified by multiplying the number of nodes in a network minus one and the mean absolute edge weight (e.g., (14-1) × 0.06 = 0.78). We argue that the standard version of the node strength could poorly identify bridge nodes when tightly connected clusters of nodes are weakly connected with each other. Therefore, we used a modified version of the node strength (Bereznowski et al., 2021). The modified version of the node strength is a metric equal to the sum of absolute values of all edges (1) a node representing dimensions of work engagement, job burnout, and perceived stress has with nodes representing symptoms of work addiction (e.g., the sum of absolute values of edges between absorption and symptoms of work addiction) or (2) a node representing a symptom of work addiction has with nodes representing other phenomena (e.g., the sum of absolute values of edges between mood modification and dimensions of work engagement, job burnout, and perceived stress). Its lowest possible value is 0.00 (a node is not connected). Its highest possible value is the number of nodes representing other phenomena in a network minus one (a node is connected with all nodes representing other phenomena in a network with edges of magnitude 1.00). The importance of a specific node is determined based on a comparison with other nodes from the same group. The modified version of the node strength should better capture bridge nodes in this special case and allow easier identification of important nodes when many edges connecting clusters are present.

To estimate the predictability of nodes, we used the mgm 1.2-11 package (Haslbeck, 2019). For continuous data (dimensions of work engagement, dimensions of job burnout, and perceived stress), node predictability indicates the percentage of variance explained by all of its neighbors (*R*2). For ordinal data (symptoms of work addiction), node predictability indicates how much a node can be predicted by all of its neighbors, beyond what is trivially predicted by the marginal distribution of this node (for a detailed explanation, see Haslbeck and Waldorp, 2018).

## 3. Results

### 3.1. Descriptive statistics

Means, standard deviations, skewness, and kurtosis of the seven symptoms of work addiction, the three dimensions of work engagement, the three dimensions of job burnout, and the general score of perceived stress are presented in Table 2.

**TABLE 2:** Skewness, kurtosis, means, and standard deviations of the study variables.

No.	Node	Skewness (kurtosis)	*M* (SD)	Number of items	Possible range
1	Salience	0.13 (2.03)	2.55 (1.13)	1	1–5
2	Tolerance	−0.34 (2.34)	3.19 (1.04)	1	1–5
3	Mood modification	0.57 (2.16)	2.20 (1.19)	1	1–5
4	Relapse	0.43 (2.07)	2.37 (1.21)	1	1–5
5	Withdrawal	0.57 (2.40)	2.25 (1.16)	1	1–5
6	Conflict	0.07 (1.89)	2.67 (1.23)	1	1–5
7	Problems	0.55 (2.15)	2.18 (1.16)	1	1–5
8	Vigor	−0.55 (2.53)	14.32 (4.20)	3	3–21
9	Dedication	−0.57 (2.54)	15.06 (4.42)	3	3–21
10	Absorption	−0.47 (2.46)	14.09 (4.50)	3	3–21
11	Exhaustion	0.72 (2.89)	15.84 (6.91)	5	5–35
12	Cynicism	1.04 (3.81)	14.71 (5.90)	5	5–35
13	Professional efficacy	−0.62 (2.91)	31.32 (6.90)	6	6–42
14	Perceived stress	0.32 (2.97)	26.51 (6.64)	10	10–50

### 3.2. Network analysis

Stability analysis showed that the network was accurately estimated, with small to moderate confidence intervals around the edge weights. The correlation stability coefficient equaled 0.67 and exceeded the recommended threshold of 0.50 for stability estimation (Epskamp et al., 2018).

**FIGURE 2:**
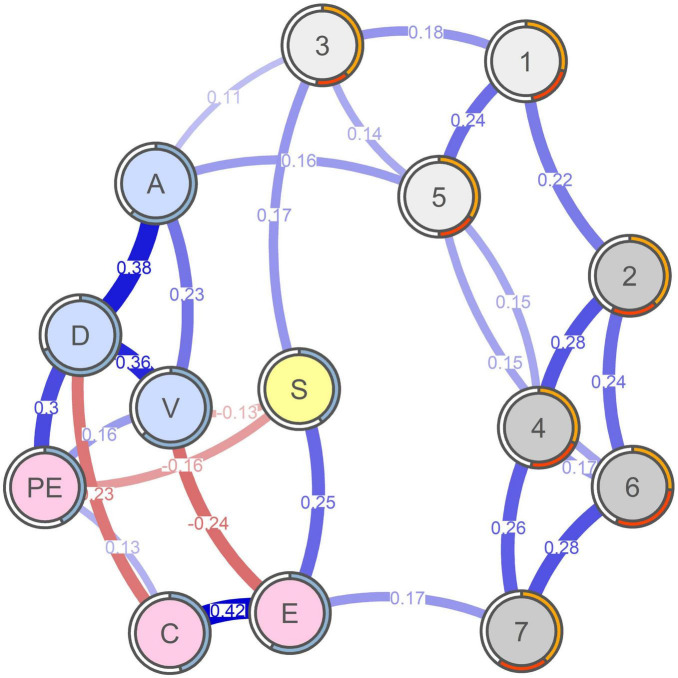
The regularized partial correlation network. The lighter gray nodes represent the less pathological symptoms of work addiction, the darker gray nodes represent the more pathological symptoms of work addiction, the yellow node represents perceived stress, the pink nodes represent the dimensions of job burnout, and the blue nodes represent the dimensions of work engagement. Blue lines represent positive edges, and red lines represent negative edges. Line thickness and darkness indicate the strength of a relationship. In the case of symptoms of work addiction, the orange area in the ring around a node represents predictability based on the variance of a symptom explained by all of its neighbors. The red area in the ring around a node represents predictability based on the marginal distribution of a node. In the case of dimensions of work engagement, the blue area in the ring around a node represents a proportion of explained variance (*R*^2^). 1 = salience; 2 = tolerance; 3 = mood modification; 4 = relapse; 5 = withdrawal; 6 = conflict; 7 = problems. S, perceived stress; E, exhaustion; C, cynicism; PE, professional efficacy; V, vigor; D, dedication; A, absorption.

The estimated network is visualized in Figure 2. The network density equaled 0.30 (27/91 edges), and the mean absolute edge weight equaled 0.06. The spin-glass algorithm showed that there were four clusters in data. The first cluster included Salience (1), mood modification (3), and withdrawal (5). The second cluster included tolerance (2), relapse (4), conflict (6), and problems (7). The third cluster included vigor (V), dedication (D), absorption (A), and professional efficacy (PE). The fourth cluster included exhaustion (E), cynicism (C), and stress (S). There were four direct edges between symptoms of work addiction and other nodes in the network: mood modification (3)—absorption (A), mood modification (3)—stress (S), withdrawal (5)—absorption (A), and problems (7)—exhaustion (E).

**FIGURE 3:**
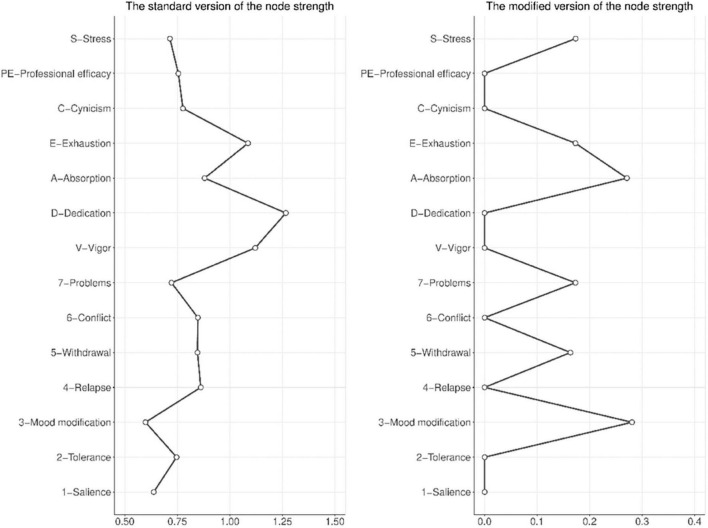
The **(left panel)** shows the unstandardized values of the standard version of the node strength in the network. The **(right panel)** shows the unstandardized values of the modified version of the node strength in the network. The modified version of the node strength is a metric equal to the sum of absolute values of all edges (1) a node representing dimensions of work engagement, job burnout, and perceived stress has with nodes representing symptoms of work addiction (e.g., the sum of absolute values of edges between absorption and symptoms of work addiction) or (2) a node representing a symptom of work addiction has with nodes representing other phenomena (e.g., the sum of absolute values of edges between mood modification and dimensions of work engagement, job burnout, and perceived stress).

The standard version of the node strength (which takes into account all edges in the network) showed that Dedication (D) was the most central node, and that Mood modification (3) was the least central node in the network (see a left panel in Figure 3). The modified version of the node strength (which takes into account only the edges with work addiction symptoms) showed, unsurprisingly, that the most central nodes were Mood modification (3) and Absorption (A; see a right panel in Figure 3).

The average predictability in the network equaled 42.0%, the average predictability for symptoms of work addiction equaled 29.9%, and the average predictability for other variables in the network equaled 54.1%. The most predictable symptom of work addiction was Conflict (6) whose predictability equaled 30.5%, and the least predictable symptom of work addiction was Mood modification (3) whose predictability equaled 13.4%.

## 4. Discussion

In this study, we aimed to investigate the direct relationships of work addiction symptoms with dimensions of work engagement, job burnout, and perceived stress among working Poles from the general population. In the estimated network, we observed four clusters of nodes. The first and second clusters included work addiction symptoms of potentially different levels of pathology. This is not a novel result as the sample used in this study was one of the samples used in previous studies on network approach to work addiction (Bereznowski et al., 2021, 2022). The third cluster included work engagement dimensions and professional efficacy, which is a dimension of job burnout, and the fourth cluster, included the two remaining job burnout dimensions and perceived stress.

While we expected most of these results, the fact that professional efficacy was in a cluster with work engagement dimensions rather than with job burnout dimensions is worth attention. Even more so, taking into account that the only direct relationship between professional efficacy and other dimensions of job burnout is a positive relationship with cynicism, which indicates that more cynical individuals are also more efficient. These results indicate that professional efficacy shares some unique variance with both work engagement dimensions (dedication and vigor) and cynicism. Consequently, they might indicate that both being passionate about one’s work as well as being cynical about it could contribute to being more professionally efficient. However, future studies should investigate whether the magnitude of the shared unique variances is sufficient for such an interpretation to be feasible, as this result seems counterintuitive. Moreover, these results support the notion that professional efficacy is not a part of burnout syndrome, which is increasingly emphasized in the literature and supported by most data (Bianchi and Schonfeld, 2018; Bianchi et al., 2019). On the other hand, professional efficacy is a positively framed construct within a negative burnout syndrome, and is measured as such. Therefore, measurement artifacts related to reverse phrasing of items (positive instead of negative inefficacy) in comparison to other burnout components (negative) need to be taken into account (Gnambs et al., 2018).

Also, it is worth noting that burnout and engagement components were closely associated and ostensibly more with each other than with work addiction. Firstly, it provides further support to the notion that engagement and burnout represent polar opposites of the same construct, which is one of the dominant conceptualizations of their relationship (Maslach and Leiter, 2016). Please note that the two negative edges were observed between dedication and cynicism and between vigor and exhaustion. Based on their definitions, it seems intuitive that an individual with a high level of dedication (enthusiasm about one’s work) has a low level of cynicism (negative attitude toward work) and an individual with a high level of vigor (high level of energy) has a low level of exhaustion (low level of energy), and vice versa. Secondly, it supports the notion that work addiction is a clearly separate phenomenon, related to both of them *via* specific pathways.

Work addiction symptoms were connected with dimensions of work engagement, job burnout, and perceived stress through four edges. Mood modification was directly related to absorption and perceived stress. Withdrawal was directly related to absorption. Problems symptom was directly related to exhaustion. These results are mostly congruent with our hypotheses (we did not observe the expected direct relationship between withdrawal and stress as well as direct relationships between tolerance, relapse, and conflict and job burnout dimensions). Based on the addiction model and engagement and burnout theories, these results suggest that engagement may turn into addiction when absorption begins to be habitually used for mood modification purposes among chronically stressed individuals experiencing negative emotional states (Maslach and Leiter, 2016; Bailey et al., 2017; Bianchi and Schonfeld, 2018; Atroszko et al., 2020a; Atroszko, 2022a,b). It may lead to withdrawal symptoms when a person cannot work and become absorbed in work. Absorption is a positive state, sometimes compared to flow, which an individual is experiencing while working (Di Stefano and Gaudiino, 2019). At the same time, an individual absorbed in work might have “difficulties with detaching oneself from work” (Schaufeli et al., 2002, p. 75). It is closely associated with the addictive process and explains the relationship between absorption and withdrawal. In turn, work addiction may lead to burnout by mounting problems leading to exhaustion.

The lack of a direct relationship between withdrawal and perceived stress is surprising. The withdrawal symptom is measured with the question, “How often during the last year have you become stressed if you have been prohibited from working?” (Andreassen et al., 2012, p. 269). Therefore, stress is a central part of this symptom’s definition, and it seems justified to expect that there exists a direct relationship between the withdrawal symptom and perceived stress. However, it is also probable that stress resulting from being prohibited from working is only a very small part of stress experienced by people in general. This difference in magnitudes of stress is responsible for the lack of an edge in the estimated network.

The lack of direct relationships between tolerance, relapse, and conflict and dimension of other phenomena is surprising as well. Especially unexpected is the lack of a relationship between tolerance and exhaustion, as spending long hours working could lead to exhaustion. However, as network analysis is focused on direct relationships between nodes, it is possible that the three symptoms are only indirectly related to job burnout dimensions and problems and exhaustion are mediators of these relationships. These findings are particularly important as they narrow down and specify hypotheses regarding potential mechanisms leading from engagement to addiction and addiction to burnout.

### 4.1. Strengths and limitations

We performed the investigation in a relatively large sample, which resulted in the stability of the estimated network. We used widely used instruments to measure work engagement, job burnout, and perceived stress. As these instruments use several items per dimension, the bias introduced by the unreliability of dimensions measurement should be reduced. The network estimated in this study included the external field of work addiction symptoms and as such, contributes to the still scant literature on the external fields of mental disorders (Borsboom, 2017; Fried, 2020).

In terms of limitations, the sample was predominantly female and represented the general population of working individuals from just one country, thus limiting the generalizability of the results to other populations. The symptoms of work addiction were measured with single items, which may influence the estimates of network parameters. Finally, the data were cross-sectional, thus causal inference is limited.

### 4.2. Conclusion, implications, and future studies directions

This study showed that burnout and engagement components were closely associated and more with each other than work addiction which supports the notion that engagement and burnout represent polar opposites of the same construct. It also strongly corroborates the assumption that work addiction is a separate phenomenon, related to both of them *via* specific pathways. The symptoms of work addiction were connected with other phenomena through four direct relationships: (1) mood modification—absorption, (2) mood modification—stress, (3) withdrawal—absorption, and (4) problems—exhaustion. Based on these, feasible mechanisms leading from work engagement to burnout through work addiction can be suggested. These focus on the absorption component and mood modification related to efforts focused on alleviating chronic stress and negative emotional states. In turn, problems arising from work addiction may lead to exhaustion. The current network analysis study provided data on feasible mechanisms leading from engagement to burnout through work addiction. These should be investigated in detail in subsequent studies, which may lead to proper prevention programs and therapeutic interventions.

Three potential interventions, which can benefit both individuals at risk of developing work addiction and individuals already addicted to work, can be proposed based on the results of this study. First, it is worth informing and instructing the individuals that it is healthier for them to alleviate the experienced stress through active problem-solving, support from friends and family, and/or mindfulness practice rather than excessive work (the relationship between perceived stress and mood modification). Second, it is crucial to teach the individuals the difference between positive absorption into work, which increases their focus and productivity during a few hours (such as flow; Schaufeli et al., 2002), and negative absorption into work, which results in negligence of other areas of life, interpersonal relationships, and other needs in the span of several days, weeks, or months (the relationships between absorption and mood modification and withdrawal). Third, it is important to educate the individuals that they can perform on a certain level only for a specific number of hours during each day and that working extra hours for longer periods leads to work-related problems, job burnout, and eventually reduced job performance (the relationship between exhaustion and problems).

Future studies should focus on further investigation of networks, including work addiction symptoms and dimensions of other work-related phenomena. The possible extensions of our work include: (1) the estimation of networks with additional work-related phenomena (e.g., work-life conflict), (2) the estimation of the network with the same phenomena measured with different instruments, or (3) the estimation of moderated network models including various work-related phenomena as moderators (Haslbeck et al., 2021). Moreover, intensive longitudinal methods might allow the investigation of direct causal relationships between work addiction symptoms and dimensions of other work-related phenomena. Last but not least, replications of this study are also highly warranted.

## Data availability statement

The original contributions presented in this study are included in the article/Supplementary material, further inquiries can be directed to the corresponding author.

## Ethics statement

The studies involving human participants were reviewed and approved by the Norwegian Data Protection Official for Research and the Research Ethics Committee at the Institute of Psychology of the University of Gdańsk in Poland. The patients/participants provided their written informed consent to participate in this study.

## Author contributions

PB assisted with obtaining funding, literature search, study design and concept, statistical analyses, data interpretation, generation of the initial draft of the manuscript, manuscript preparation and editing, final editing, and approval of the manuscript. PA assisted with the literature search, study design and concept, data collection, data interpretation, manuscript preparation and editing, final editing, and approval of the manuscript. RK assisted with the study design and concept, manuscript preparation and editing, final editing, and approval of the manuscript. All authors contributed to the article and approved the submitted version.
